# National and sub-national sero-epidemiology of immunoglobulin G against SARS-CoV-2 in Iran in 2021

**DOI:** 10.1371/journal.pone.0313795

**Published:** 2025-07-31

**Authors:** Mohammadreza Azangou-Khyavy, Erfan Ghasemi, Narges Ebrahimi, Mohammad-Mahdi Rashidi, Mohsen Abbasi-Kangevari, Naser Ahmadi, Javad Khanali, Ameneh Kazemi, Arezou Dilmaghani-Marand, Yosef Farzi, Moein Yoosefi, Elham Abdolhamidi, Mana Moghimi, Maryam Nasserinejad, Nima Fattahi, Sina Azadnajafabad, Arefe Alipour-Daroei, Kamyar Rezaee, Shirin Djalalinia, Negar Rezaei, Hamidreza Jamshidi, Farshad Farzadfar

**Affiliations:** 1 Non-Communicable Diseases Research Center, Endocrinology and Metabolism Population Sciences Institute, Tehran University of Medical Sciences, Tehran, Iran; 2 Endocrinology and Metabolism Research Center, Endocrinology and Metabolism Clinical Sciences Institute, Tehran University of Medical Sciences, Tehran, Iran; 3 Faculty of Medicine, Center for Life Course Health Research, University of Oulu, Oulu, Finland; 4 Department of Internal Medicine, Yale School of Medicine, New Haven, Connecticut, United States of America; 5 Deputy of Research and Technology, Ministry of Health and Medical Education, Tehran, Iran; 6 Department of Pharmacology, Research Institute for Endocrine Sciences, School of Medicine, Shahid Beheshti University of Medical Sciences, Tehran, Iran; World Health Organization, EGYPT

## Abstract

Multiple factors challenge PCR test results for COVID-19 infection, and only symptomatic cases have been tested. Thus, a population-based seroprevalence study was necessary to determine the extent of missed cases. The objective of this study was to achieve a realistic infection rate in Iran and probe into some explanations behind being infected or not. In this population-based cross-sectional study, 16,610 adults aged more than 25 with valid serology sample results from 31 provinces from February to April 2021 were included. According to the ELISA kits based on the N antigen of SARS-CoV-2, the seroprevalence of IgG against SARS-CoV-2 in Iran was 20.63% (19.71–21.56) and 16.25% (15.11–17.41) based on two different corrections. The age-standardized seroprevalence was relatively high among Kurdistan [30.29% (26.04–34.55) and 28.31% (23–33.61)] and West Azarbayejan [29.33% (24.85–33.8) and 27.11% (21.52–32.68)]. Smoking, higher education, being underweight, male, and single were protective factors, and higher daily interactions was a risk for seropositivity. It is evident that reported infection rates have been misleading. Furthermore, several intervenable factors can predict the risk of infection.

## 1 Introduction

COVID-19 was first reported in the Wuhan province of China and has been spreading across the globe ever since [1]. Without determining the number of infected people, it is impossible to implement cost-effective measures to halt the virus spread. Nevertheless, determining the actual infection rates has remained challenging for health systems due to the high proportion of asymptomatic infected individuals [2,3].

COVID-19 diagnosis is primarily based on reverse-transcription polymerase chain reaction (RT–PCR), where the test result is sensitive to multiple factors, including specimen collection, transportation, and storage methods that might affect the test’s sensitivity [2,4,5]. Besides, the use of PCR testing for population-based studies is challenged by some countries’ preferred approaches to test only symptomatic or suspected cases due to limited resources [6].

The anti-SARS-CoV-2 antibodies in serum samples would be positive even if the individual had been asymptomatic [7]. Although both anti-SARS-CoV-2 immunoglobulin G (IgG) and M (IgM) could be measured to determine the infection history of an individual, IgG tends to remain higher for more extended periods [8]. The IgG is more likely to be detected in participants’ serum samples in a cross-sectional study. Hence, population-based seroprevalence studies are required to achieve the proportion of infected and not infected people and probe into some possible explanations behind being infected or not. Such results would also be valuable for investigating the effectiveness of measures taken by governments and people in mitigating the infection rate for future waves of pandemics before the availability of vaccines.

Indeed, population-based seroepidemiological data would shed light on the extent of past infections and immune responses, aiding in the formulation of evidence-based strategies for public health interventions. Hence, the objective of this study was to determine the seroprevalence of IgG against SARS-CoV-2 and its sociodemographic, medical, and behavioral determinants in Iran. Since the samples for this study were recruited before the availability of anti-COVID-19 vaccines in the country, this population-based seroprevalence study provides insights into realistic infection rates in Iran at the national and provincial levels.

## 2 Materials and methods

### 2.1 Study design

This population-based cross-sectional study was approved by the Ethical Committee of the National Institute for Health Research under reference number IR.TUMS.NIHR.REC.1398.006. The epidemiologic and serological data were extracted from the STEP-wise approach to non-communicable disease risk factor surveillance (STEPS) 2021 survey. The complete timeline and study design are available in the supplements. STEPS 2021 data was gathered after a systemic cluster classification method of sampling and through three steps of filling questionnaires, physical measurements, and lab tests. This second-hand data was anonymously processed. Hence, informed consent was not applicable. However, participation in the STEPS 2021 survey was voluntary, and all participants provided written informed consent. More details regarding sampling methods and study protocol of STEPS 2021 are defined explicitly elsewhere [9]. The calculated age-standardized seroprevalences at national and sub-national levels were corrected by the test’s sensitivity and specificity. Finally, the association of sociodemographic variables, behaviors, past medical history, and compliance to COVID-19 safety protocols with the IgG seropositivity were investigated.

### 2.2 Study settings, size, and participants

The study was conducted with the participation of 27,874 adults aged more than 18 from 31 provinces of Iran in 2021. The final study population was 16,610 individual-level valid serological sample results that were considered IgG positive or negative and were 25 and above.

### 2.3 Variables

The variables of this study included IgG test results; sociodemographic variables including age, sex, education, occupation, marital status, quality of life, and wealth index; past medical history including diabetes mellitus, hypertension, hypercholesterolemia, myocardial infarction, lung disease, stroke, cancer, and kidney disease; behavioral variables including current cigarette smoking, second-hand smoking, physical activity (i.e., Metabolic Equivalent of Task [MET]), and alcohol consumption; BMI; COVID-19 safety protocols compliance including wearing masks, face shields, gloves, and washing hands (S2 Table in S1 File). The variables such as education, occupation, marital status, past medical history, behaviors, and safety protocols compliance were assessed based on self-reported questionnaires in 2021. A detailed description of the quality of life, wealth index, MET, and alcohol consumption is in the supplementary material on page 2.

### 2.4 Data source

STEPS survey, initially introduced by the World Health Organization, is a standardized and straightforward method for collecting and analyzing data on NCDs’ risk factors across a country. It can include a wide range of topics [10] and has been conducted in Iran in 2005−2009, 2011, and 2016 [11]. In the 2021 survey, the data was gathered from February 2021 to April 2021 (S1 Table in S1 File) in Iran. As anonymously processed second-hand data (STEPS registered data) were used, consent was not required. However, written informed consent was obtained from participants in each round of the STEPS study. The data of 27874 individuals were recruited from the registry database through consecutive processes of filling out questionnaires, getting physical measurements, and doing a lab examination. The sampling strategy used was systematically proportional to size cluster random sampling. Following some changes in the original project introduced by the World Health Organization, due to the COVID-19 pandemic, the anti-SARS-CoV-2 IgG ELISA tests have been run on serum samples collected from participants, and COVID-19 related variables, including compliance with preventive measures, were studied.

### 2.5 Statistical analysis

#### 2.5.1 Serum IgG measurement and seroprevalence correction.

IgG serum level assessments were conducted via a SARS-CoV-2 ELISA diagnostic kit provided by Pishtaz Teb Diagnostics with the basis of the N antigen of SARS-CoV-2 [12] (supplementary material, page 2), approved by Iran’s Food and Drug Administration. After determining each sample’s optical density and the cut-off value according to the manufacturer’s instructions, samples were categorized as IgG positive or negative. However, the ELISA kit has a particular sensitivity and specificity that have to be considered while determining the real seroprevalence. In this regard, the seroprevalence has to be corrected by the test’s sensitivity and specificity. To calculate the corrected prevalence of seropositive individuals, crude prevalence, the test’s sensitivity, and specificity were inserted into the below formula [13]:


$$Corrected\;seroprevalence=\frac{crude\;seroprevalence-(1-specificity)}{sensitivity-(1-specificity)}$$

This ELISA kit’s sensitivity and specificity were determined both by the provider and an external laboratory. The provider-reported (first correction) sensitivity and specificity of these kits were 94.1% and 98.3%, respectively. Iran’s Pasteur Institute further evaluated the kits’ specs to validate them (second correction) and their reported sensitivity and specificity were 82.8% and 91.28%, respectively.

#### 2.5.2 Odds ratios.

To investigate possible associations between all of the abovementioned variables and IgG seropositivity, the Odds Ratios (ORs) and P values were calculated. The crude ORs were calculated in addition to the adjusted ORs by age group, sex, education, and wealth index. The seroprevalence by the second correction was only used to calculate ORs. Furthermore, COVID-19 infection history based on participants’ claims was also considered for calculating ORs of study variables and infection history (S4 Table in S1 File). All of the results of seroprevalence and ORs are reported with a 95% Uncertainty Interval (UI) at national and sub-national level based on the total sample size and sample size of each province. The statistical analyses and visualizations were carried out using R statistical packages v4.1.2 (http://www.r-project.org, RRID: SCR_001905) (S1 Fig in S1 File).

## 3 Results

### 3.1 Main results

#### 3.1.1 SARS-CoV-2 seroprevalence.

**Fig 1:**
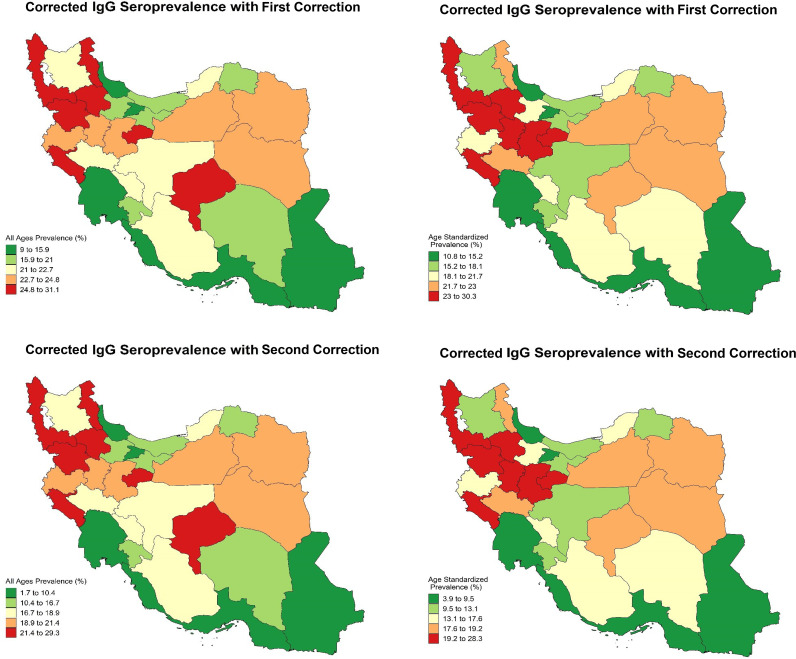
Age-standardized and all-ages seroprevalence of IgG against SARS-CoV-2 in Iran at the subnational level, adjusted by first and second corrections.

The seroprevalence of IgG against SARS-CoV-2 in Iran at the national level was 20.63% (19.71–21.56) and 16.25% (15.11–17.41) based on the first and second corrections, respectively (Table 1). At the province level, there were disparities in the seroprevalence of IgG. The highest seroprevalence was in Kurdistan with 30.29% (26.04–34.55) by the first and 28.31% (23–33.61) by the second correction, and the lowest seroprevalence was in Hormozgan with 10.75% (6.76–14.73) by the first and 3.93% (0–8.9) by the second correction (Table 1 and Fig 1).

**Table 1:** Age-standardized seroprevalence of IgG against SARS-CoV-2 in Iran at national and subnational levels by first and second corrections.

Location	Sample size	N	Crude prevalence % (95% UI))	Prevalence by first correction % (95% UI)	Prevalence by second correction % (95% UI)
**National**	16610	3598	20.76% (19.91-21.62)	20.63% (19.71-21.56)	16.25% (15.11-17.41)
**Alborz**	237	36	15.36% (9.07-21.66)	14.78% (7.98-21.6)	8.96% (0.47-17.47)
**Ardebil**	423	112	22.21% (17.84-26.59)	22.2% (17.47-26.94)	18.21% (12.31-24.12)
**Azarbayejan-East**	483	108	18.24% (14.37-22.12)	17.9% (13.71-22.1)	12.85% (7.63-18.09)
**Azarbayejan-West**	538	169	28.8% (24.66-32.93)	29.33% (24.85-33.8)	27.11% (21.52-32.68)
**Bushehr**	515	64	11.92% (8.88-14.97)	11.06% (7.77-14.36)	4.32% (0.22-8.44)
**Chahar Mahaal and Bakhtiari**	478	108	21.65% (17.52-25.79)	21.59% (17.12-26.07)	17.45% (11.88-23.04)
**Fars**	870	196	21.68% (18.52-24.84)	21.62% (18.2-25.04)	17.49% (13.23-21.76)
**Gilan**	372	59	13.62% (9.62-17.62)	12.9% (8.57-17.23)	6.61% (1.21-12.01)
**Golestan**	524	118	21.1% (17.45-24.76)	21% (17.05-24.96)	16.71% (11.78-21.65)
**Hamedan**	570	138	22.98% (19.01-26.94)	23.03% (18.73-27.32)	19.25% (13.89-24.6)
**Hormozgan**	365	38	11.63% (7.95-15.31)	10.75% (6.76-14.73)	3.93% (0-8.9)
**Ilam**	360	103	27.94% (22.88-32.99)	28.4% (22.92-33.86)	25.94% (19.11-32.76)
**Isfahan**	581	125	18.36% (14.82-21.91)	18.03% (14.2-21.87)	13.01% (8.23-17.81)
**Kerman**	376	60	18.43% (13.37-23.49)	18.11% (12.63-23.58)	13.11% (6.28-19.94)
**Kermanshah**	404	91	20.69% (15.78-25.6)	20.55% (15.24-25.87)	16.16% (9.53-22.79)
**Khorasan-North**	578	105	18.14% (14.75-21.53)	17.79% (14.12-21.46)	12.72% (8.14-17.29)
**Khorasan-Razavi**	1001	246	22.56% (19.71-25.4)	22.58% (19.49-25.65)	18.68% (14.84-22.52)
**Khorasan-South**	510	121	22.16% (18.08-26.23)	22.14% (17.73-26.55)	18.14% (12.63-23.64)
**Khuzestan**	597	84	12.4% (9.6-15.19)	11.58% (8.55-14.6)	4.97% (1.19-8.73)
**Kohgiluyeh and Boyer-Ahmad**	492	82	15.76% (12.4-19.13)	15.22% (11.58-18.86)	9.5% (4.97-14.05)
**Kurdistan**	579	181	29.69% (25.76-33.62)	30.29% (26.04-34.55)	28.31% (23-33.61)
**Lorestan**	604	136	21.73% (18.13-25.34)	21.68% (17.78-25.58)	17.56% (12.7-22.44)
**Markazi**	485	124	23.23% (18.68-27.77)	23.3% (18.38-28.21)	19.59% (13.44-25.72)
**Mazandaran**	514	91	17.08% (13.13-21.04)	16.65% (12.37-20.93)	11.29% (5.95-16.63)
**Qazvin**	415	86	19.36% (15.11-23.62)	19.11% (14.51-23.72)	14.36% (8.63-20.11)
**Qom**	413	96	24.8% (19.06-30.54)	25% (18.79-31.21)	21.71% (13.96-29.45)
**Semnan**	451	99	22.13% (17.17-27.1)	22.11% (16.74-27.49)	18.1% (11.41-24.81)
**Sistan and Baluchistan**	609	99	15.56% (12.29-18.82)	15% (11.46-18.53)	9.23% (4.82-13.63)
**Tehran**	1099	237	17.23% (14.68-19.78)	16.81% (14.05-19.57)	11.49% (8.05-14.93)
**Yazd**	387	91	22.07% (16.54-27.6)	22.05% (16.06-28.03)	18.02% (10.56-25.49)
**Zanjan**	780	195	24.48% (21.11-27.85)	24.65% (21.01-28.3)	21.27% (16.73-25.82)

### 3.2 Other analyses

#### 3.2.1 IgG seroprevalence association with sociodemographic factors.

**Fig 2:**
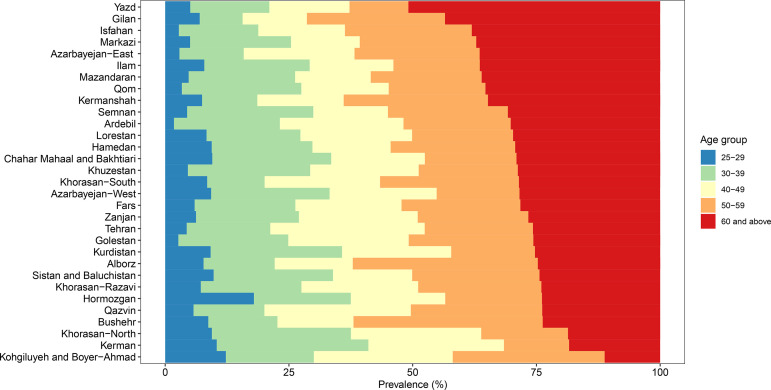
Age distribution of IgG seropositive individuals at the subnational level in Iran.

In the country, the individuals over 60 years old had higher IgG seroprevalence with 22.93% (20.28–25.59) by the second correction (OR: 1.599 [1.251–2.045], P < 0.001). Age distribution of IgG seropositive individuals at the subnational level is demonstrated in Fig 2.

**Fig 3:**
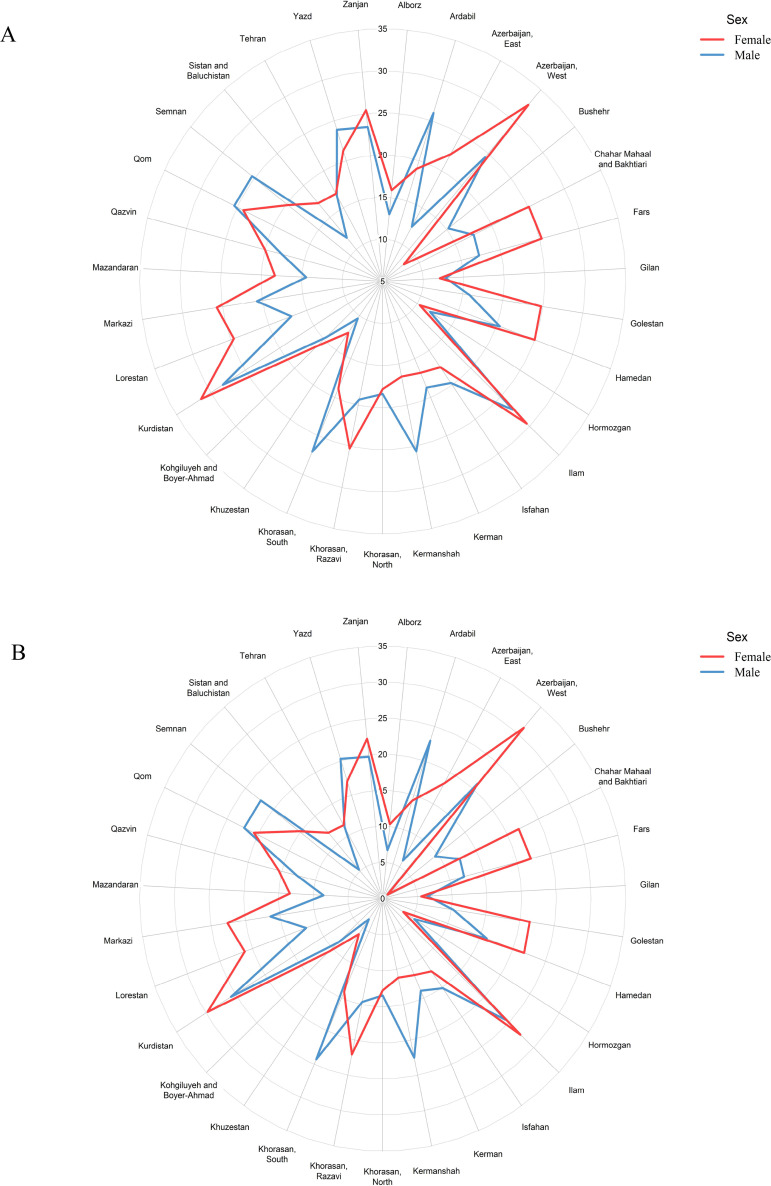
Sex distribution of IgG seropositive individuals at the subnational level, A: adjusted by first, B: adjusted by the second correction.

**Fig 4:**
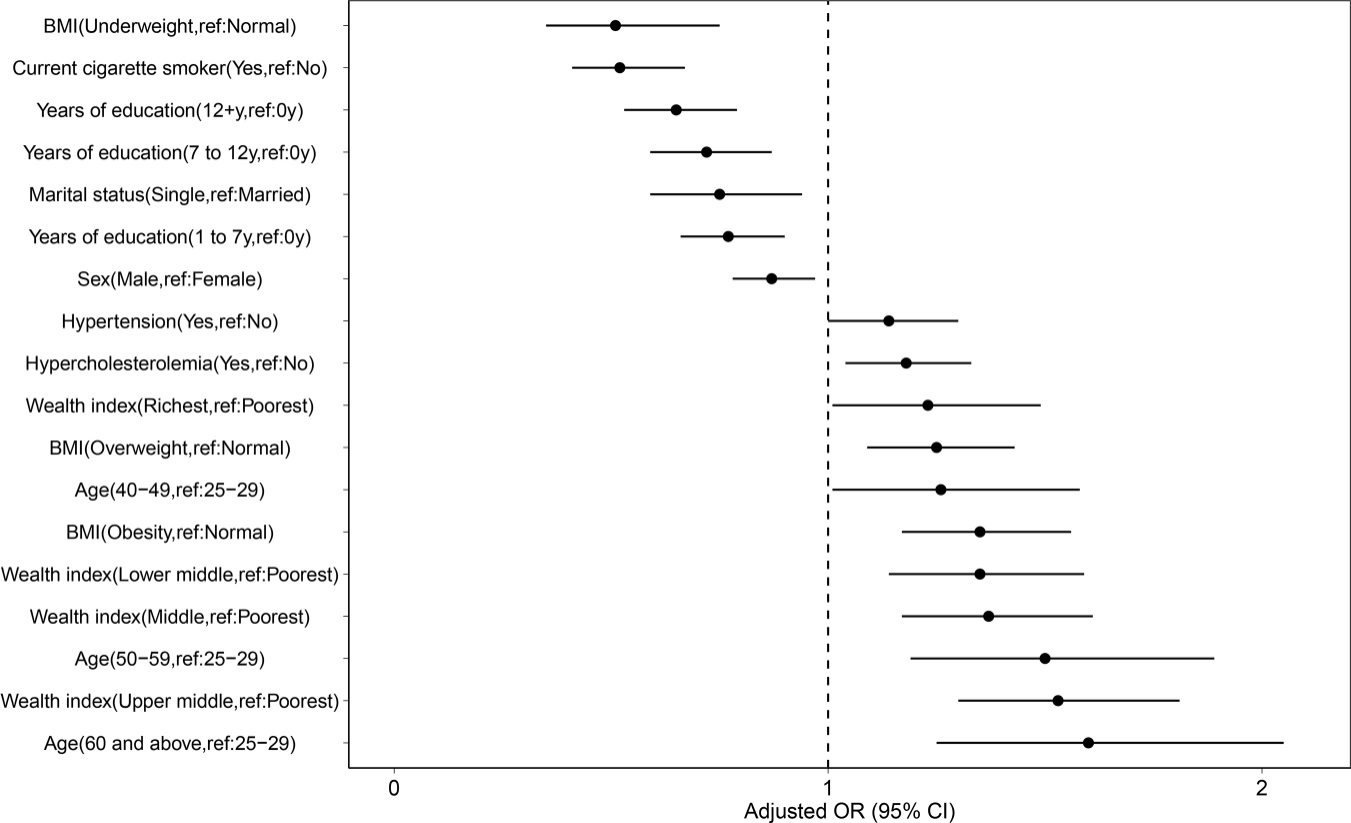
The protective and risk factors of IgG seropositivity based on OR.

Moreover, men had lower IgG seroprevalence than women 14.28% (12.59–15.97) Vs 17.85% (16.28–19.41) by the second correction (OR: 0.867 [0.775–0.969], P = 0.012). The sex distribution of IgG seropositive individuals at the subnational level is demonstrated in Fig 3. Participants with higher years of education had lower IgG seroprevalence as well. The IgG seroprevalence among the participants with over 12 years of education was 11.83% (9.89–13.74) by the second correction (OR: 0.651 [0.533–0.793], P < 0.001). Furthermore, participants who were single had lower IgG seroprevalence, which was 6.47% (3.27–9.67) by the second correction (OR: 0.747 [0.593–0.942], P = 0.014). The upper-middle wealth index, on the other hand, was associated with higher IgG seroprevalence with 19.02% (16.32–21.71) by the second correction (OR: 1.534 [1.299–1.812], P < 0.001) (S2 and S3 Tables in S1 File, Fig 4).

#### 3.2.2 IgG seroprevalence association with comorbidities.

Among the comorbidities including diabetes mellitus, hypertension, hypercholesterolemia, myocardial infarction, stroke, lung, kidney disease, and cancer history, in comparison with the reference groups (i.g., without diabetes mellitus), hypertension and hypercholesterolemia had the lowest P values of ORs. Accordingly, the IgG seroprevalence among participants with hypercholesterolemia was 21.26% (19.02–23.5) by the second correction (OR: 1.182 [1.044–1.339], P = 0.008), and the seroprevalence among participants with hypertension was 21.53% (19.47–23.6) by second correction (OR: 1.143 [1.004–1.3], P = 0.043) (S2 and S3 Tables in S1 File, Fig 4).

#### 3.2.3 IgG seroprevalence association with behavioral factors and BMI.

Among the behavioral risk factors, including current cigarette smoking, frequency of alcohol consumption, physical activity, and oral hygiene, the only factor with a significant effect on seroprevalence was current cigarette smoking (OR: 0.528 [0.41–0.679], P < 0.001).

Regarding the BMI, the underweight participants had lower, and overweight and obese participants had higher IgG seroprevalence than normal-BMI participants. The IgG seroprevalence among underweight participants was 0.58% (0–4.59) by the second correction (OR: 0.505 [0.344–0.741], P < 0.001) and The IgG seroprevalence among overweight and obese participants were 17.16% (15.24–19.07) and 20.95% (18.67–23.23) by the second correction, respectively (overweight OR: 1.234 [1.075–1.418], P = 0.003, obese OR: 1.333 [1.153–1.542], P < 0.001) (S2 and S3 Tables in S1 File, Fig 4).

#### 3.2.4 IgG seroprevalence association with COVID-19 safety protocols compliance.

COVID-19 safety protocols studied here were wearing face masks, shields, gloves, washing hands, and the number of daily contacts. Face shields and the number of daily contacts were the factors having the lowest P values. The seroprevalence of IgG among participants wearing face shields was 7.09% (2.65–11.51) by the second correction (OR: 0.692 [0.517–0.925], P = 0.013). The IgG seroprevalence among participants with the highest number of daily contacts (level four) was 19.69% (13.86–25.53) by the second correction (OR: 1.44 [1.102–1.882], P = 0.008) (S2 and S3 Tables in S1 File, Fig 4).

## 4 Discussion

The seroprevalence of IgG against SARS-CoV-2 in Iran was 20.63% and 16.25% at the time of study based on the first and second corrections. At the province level, the age-standardized seroprevalence was relatively high among Kurdistan, West Azarbayejan, and Ilam, all of which were outskirt provinces on the west, possibly due to higher connections with neighboring countries. According to our results, male gender, smoking, higher education years, being underweight, and being single were protective factors in COVID-19 seropositivity.

Seroprevalence studies used to be conducted during the pandemic around the world to reach more realistic infection rates, vaccine efficacy, and predict the timeline of the pandemic [14,15]. In support of this study’s findings, the common point of the seroprevalence studies is the fact that the infection rates based on viral tests could be highly misleading [16]. Indeed, according to this study’s findings, even in the second correction, the number of affected people by COVID-19 in Iran would be approximately 13,650,000, which is more than five times greater than the PCR-based registry report at the time of the study [1]. However, it is worth re-mentioning that individuals under 25 years old were not included in this study. At that time, no one had received any vaccines. Hence, adjusted IgG positive results was equal to COVID-19 infection history in Iran. However, this number would even be higher if the IgG levels remained high among everyone after infection, which it does not [8,17]. Based on previous study estimates, up to the end of March 2021, the seroprevalence in Iran was 16.95 (12.91–21.01), which was higher than the global estimates and also greater than the seroprevalence in the western Asia region [18]. Our study’s findings are consistent with these estimates based on the second correction. The reported seroprevalence here in Iran was higher than in the United States, Brazil, and China, and it was lower than in India, Russia, and South Africa [18].

Regarding the sociodemographic factors, in the present study, the male gender was a protective factor regarding COVID-19 seropositivity. However, males had higher seroprevalence in most countries worldwide due to higher daily interactions [18,19]. Nevertheless, there were some countries where there were no differences between male and female seropositivity rates [20]. Although the significance of the difference remains discussable, a previous study by Poustchi et al. reported lower seroprevalence among males in Iran [12]. Possible etiologies explaining the male gender being a protective factor need to be investigated in future studies. Furthermore, in accordance with previous studies, the seroprevalence increases with age [21]. However, ages older than 65 appeared to be less susceptible to SARS-CoV-2 IgG seropositivity due to lower social interactions in those studies. The results of this study do not support this explanation, probably because of high familial loyalty bonds in the culture of Iranian people preventing the elderly from being isolated. However, the debilitation of immune responses in the case of antibody titers by aging could be a probable explanation. A higher wealth index was a risk, and higher education years were protective for SARS-CoV-2 seropositivity. In accordance, higher wealth status had a positive correlation with COVID-19 seropositivity at the global level [22]. One explanation supporting this finding might be the lower social network and person-to-person interactions among economically disadvantaged people [23]. Expectedly, higher education years was negatively associated with COVID-19 seropositivity, which might be reflecting higher preventive practices among educated people [24]. Plus, being single was another protective factor for COVID-19, in accordance with another study [24]. Indeed, single people are more isolated and are not exposed to the risk of transmission through a higher number of household members [25,26].

Regarding the comorbidities, hypertension and hypercholesterolemia were positively associated with IgG seropositivity among past medical history items. Both can be a risk of seropositivity due to their association with high BMI. On the other hand, hypertension and hypercholesterolemia are risk factors for developing more severe illnesses [27,28]. Acknowledging that the higher severity of the disease is associated with higher antibody response [29], it might be more likely to detect seropositive samples among people with hypertension and hypercholesterolemia.

Regarding the behavioral risk factors, smoking cigarettes was one of the protective factors in this study. This finding was consistent with previous studies finding smoking as a protective factor for COVID-19 infection [30,31]. This finding is not solely supported by observational studies. Indeed, the protective effect of smoking on COVID-19 infection has also been supported by a cohort study in England [32]. Although the biochemical mechanism behind this finding remains unknown, there are some possible hypotheses, including the non-specific effects of nicotine or other several chemical agents in tobacco smoke. Another hypothesis was the down-regulation of Angiotensin Converting Enzyme (ACE) receptors as the binding site of SARS-CoV viruses as a result of smoking [33–35]. However, there are several controversies about these deductions, and none of them truly justifies the protective effect of smoking against COVID-19 [36]. For example, smoking cigarettes can increase the risk of long COVID-19, post-COVID-19 syndrome, and more severe symptoms [37,38]. In addition, smoking remained a potent risk factor for all-cause mortality even in the previous cohort studies investigating the protective effect of it against COVID-19 [39]. All in all, although some studies support our findings regarding the protective effect of smoking against COVID-19 infection, most of the literature is in contrast with this finding [40]. These differences can be explained by different study methods and settings. Therefore, before rushing to any conclusions, further specific studies based on pooled data from all around the world are required to come to more precise deductions regarding smoking’s effect on COVID-19 infection [41]. In the case of BMI, underweight people were at lower while overweight and obese people were at higher risk of COVID-19 seropositivity than normal-weight people. Higher BMI was also considered a risk factor for COVID-19 in the previous studies [24,30,42]. Malnutrition and low hygiene accounted for higher infection risk among underweight people, but this just is mostly in low-income countries, and in Iran, it does not appear to be true [43].

Eventually, all of these findings can be evaluated through the duration of the “remaining seropositive” point of view. Indeed, an individual could be infected but might not remain seropositive. Thus, we also analyzed the ORs between all variables with the COVID-19 infection claims by the participants to cross-validate our results (S4 Table in S1 File). Most of the found associations of this analysis supported the associations with IgG seropositivity, which could also demonstrate the extent of validity of self-reports in this study.

Finally, among the COVID-19 safety protocols compliance variables, the high number of daily contacts was positively associated with COVID-19 seropositivity, an expected result. In contrast, protocols such as wearing masks and washing hands did not have any associations. This paradox might be explained by different definitions of participants wearing masks or washing hands or people’s different attitudes towards adherence to wearing masks [44]. For example, they might be wearing masks while driving alone but might also eat out at a crowded place not wearing one.

To the best of our knowledge, this is the first and the most comprehensive study determining the seroprevalence of IgG against SARS-CoV-2 in Iran through a nationwide and population-based study [12,45–48]. Moreover, the associations between sociodemographic variables, behaviors, past medical history, and compliance to COVID-19 safety protocols with the IgG seropositivity have been investigated using the STEPS 2021 survey in Iran. Identifying associated factors with COVID-19 infection could help targeted rising awareness efforts and resource allocation. Therefore, this study can be used as a landmark for future retrograde studies to determine the possible reasons for seroprevalence variabilities across the country. Such results would also be valuable for investigating the effectiveness of measures taken by governments and people in mitigating the infection rate for future waves of pandemics caused by resistant mutants.

Using the STEPS to study seroprevalence has many other advantages. First, the data in the STEPS survey is weighted by sociodemographics. Hence, biased standard errors are avoided [49]. Second, the high number of participants reduces the selection bias and makes the reported results more valid. We also acknowledge the limitations of the study. Being cross-sectional and observational were two limitations of this study. Therefore, the reported associations could be due to several underlying factors that were not noticeable in this study, which might explain some of the controversial findings. Moreover, many people might have been affected by COVID-19, but their antibody titer was not high at the study time. This antibody titer duration might have confounded the results of this study. However, we have alternatively analyzed the associations with COVID-19 infection self-reports to reduce the effect of this limitation. In addition, the ELISA kits utilized in this study exhibited sensitivity and specificity, which we adjusted our estimations by them. Furthermore, the variations in assay procedures, reagent quality, and operator techniques could introduce inconsistencies in results.

## 5 Conclusion

Based on this study, the COVID-19 infection rate in Iran was much higher than the PCR-based registry reports at the time of the study. Hence, it is evident that reported rates have been misleading. Furthermore, several factors can predict the risk of infection for individuals in society. These factors range from the province of residence to marital status.

## Supporting information

S1 File Supplementary appendix to the national and sub-national sero-epidemiology of immunoglobulin G against SARS-CoV-2 in Iran in 2021. (DOCX)
