# Supplementary material for: National and sub-national sero-epidemiology of immunoglobulin G against SARS-CoV-2 in Iran in 2021
Source: PLoS One. 2025 Jul 31;20(7):e0313795. doi: 10.1371/journal.pone.0313795 (PMC12312912; doi:10.1371/journal.pone.0313795)
Supplement: S1 File — (DOCX) [file pone.0313795.s001.docx]

**Supplementary Appendix to the National and Sub-National Sero-Epidemiology of Immunoglobulin G against SARS-CoV-2 in Iran in 2021**

**Timeline**

The sera of participants for measuring IgG against SARS-CoV-2 was recruited from February 2021 to April 2021.

Table S1: the STEPS 2021 survey timeline

| Questionnaire and software preparation | September 2019 – November 2019 |
| --- | --- |
| Training and equipping the team | December 2019 |
| Data recruitment before the COVID-19 pandemic | January 2020 – February 2020 |
| Adjusting the project to the pandemic situation | September 2020 – November 2020 |
| Training and equipping the team | December 2020 – January 2021 |
| Data recruitment during the COVID-19 pandemic | February 2021 – April 2021 |
| Data cleaning and analysis | May 2021 – July 2021 |

**Statistical Analysis Plan (SAP)**

The sera were recruited from the participants and the ELISA assay was run. The SARS-CoV-2 IgG-containing individuals were considered seropositive after determining the cut point. After that, the ORs were calculated between all of the selected variables and IgG seropositivity, and the P<0.05 was considered a significant association. The ORs were adjusted by age group, sex, education, and wealth index and reported.

Figure S1: the SAP of the study including the inputs, processing methods, and outputs

**
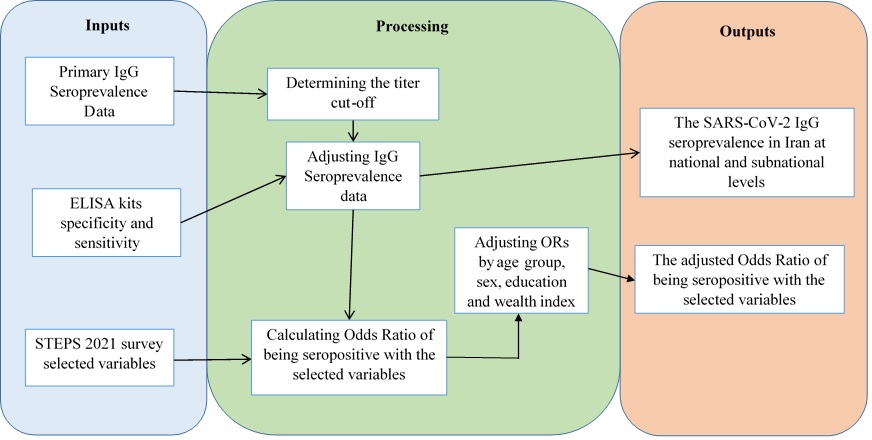
**

**Testing method**

Samples were recruited and stored in EDTA-coated tubes. The sera were centrifuged and after that, following the manufacturer’s instructions, the IgG against SARS-CoV-2 were assessed using Iran’s FDA-approved ELISA kit (Pishtaz Teb SARS-CoV-2 ELISA kits) (1). The basis of this test is the N antigen of SARS-CoV-2 which is attached to the tubes. The sera samples are added to them and if the sera contain anti-SARS-CoV-2 antibodies (i.e., IgG), it will bind to the antigen. Subsequently, IgG will be labeled and after washing, the OD will be determined at 450 nm.

**Variables definition**

**Quality of life:** the quality of life in this study is measured using the EuroQol five-dimension (EQ-5D) 3L version questionnaire (2). This questionnaire results in a score ranging from 0 to 100 which in this study is divided into five quantiles.

**Wealth index:** The wealth index is an instrument that measures the economic status of households calculated based on assets by using principal component analysis. The assets include the material of the house, type of possession of the residence, having a car or not, having how many cars, the type of car, house facilities including piped water, piped gas, electricity, bathroom, landline phone, kitchen, air conditioner, heating system, internet, type of television, refrigerator, freezer, oven, vacuum cleaner, washing machine, dishwasher, dryer machine, personal computer, laptop, mobile phone, microwave oven, and the type of fuel used for various purposes.

**Metabolic Equivalent of Task (MET):** The MET score is calculated from the Global Physical Activity Questionnaire (GPAQ) used in WHO STEPS methods to evaluate the physical activity in a person (3). One MET means 1 kcal/kg/hour which is the energy cost of sitting quietly; while Four METs are assigned to the time spent doing moderate and 8 METs are assigned to the time spent doing vigorous intensity activity. In this study, the MET variable is defined as whether or not an individual has any combination of physical activities per week that exceeds 600 METs.

**Alcohol consumption:** the amount of alcohol consumption in each frequency is defined as six glasses of drink per time.

**Table S2. The crude and corrected seroprevalences IgG among the different statuses of study variables**

| Variable | Status | N | Frequency % (95% UI) | Crude prevalence (95% UI) | Prevalence by first correction (95% UI) | Prevalence by second correction (95% UI) |
| --- | --- | --- | --- | --- | --- | --- |
| Sociodemographic |  |  |  |  |  |  |
| Age |  |  |  |  |  |  |
|  | 25-29 | 1390 | 8.57% (7.95 - 9.18) | 15.07% (12.72 - 17.42) | 14.47% (11.93 - 17.01) | 8.57% (5.4 - 11.74) |
|  | 30-39 | 3978 | 24.56% (23.6 - 25.53) | 16.29% (14.74 - 17.84) | 15.79% (14.11 - 17.47) | 10.22% (8.13 - 12.31) |
|  | 40-49 | 3968 | 23.19% (22.29 - 24.09) | 20.05% (18.31 - 21.8) | 19.86% (17.98 - 21.75) | 15.29% (12.95 - 17.66) |
|  | 50-59 | 3405 | 20.07% (19.22 - 20.93) | 23.67% (21.73 - 25.62) | 23.78% (21.68 - 25.89) | 20.18% (17.56 - 22.81) |
|  | 60 and above | 3869 | 23.61% (22.66 - 24.56) | 25.71% (23.74 - 27.68) | 25.98% (23.85 - 28.12) | 22.93% (20.28 - 25.59) |
| Sex |  |  |  |  |  |  |
|  | Female | 9424 | 55.49% (54.39 - 56.58) | 21.94% (20.78 - 23.1) | 21.9% (20.65 - 23.16) | 17.85% (16.28 - 19.41) |
|  | Male | 7186 | 44.51% (43.42 - 45.61) | 19.3% (18.05 - 20.55) | 19.05% (17.69 - 20.4) | 14.28% (12.59 - 15.97) |
| Years of education |  |  |  |  |  |  |
|  | 0 y | 2929 | 14.63% (13.97 - 15.28) | 27.73% (25.57 - 29.88) | 28.17% (25.83 - 30.5) | 25.66% (22.75 - 28.56) |
|  | 1 to 7 y | 4615 | 25.4% (24.5 - 26.29) | 22.71% (21.16 - 24.26) | 22.74% (21.06 - 24.42) | 18.88% (16.79 - 20.98) |
|  | 7 to 12 y | 3054 | 19.15% (18.31 - 19.99) | 19.65% (17.8 - 21.5) | 19.43% (17.42 - 21.43) | 14.75% (12.26 - 17.25) |
|  | 12+y | 5896 | 40.83% (39.72 - 41.93) | 17.48% (16.05 - 18.9) | 17.08% (15.53 - 18.61) | 11.83% (9.89 - 13.74) |
| Occupation |  |  |  |  |  |  |
|  | Freelancer | 3846 | 24.31% (23.37 - 25.26) | 18.83% (17.2 - 20.46) | 18.54% (16.77 - 20.3) | 13.65% (11.45 - 15.85) |
|  | Private sector worker | 972 | 6.65% (6.09 - 7.21) | 15.82% (12.63 - 19.02) | 15.28% (11.83 - 18.74) | 9.58% (5.28 - 13.9) |
|  | Public sector worker | 1176 | 7.41% (6.75 - 8.06) | 17.75% (14.73 - 20.76) | 17.37% (14.1 - 20.63) | 12.19% (8.11 - 16.25) |
|  | Retired | 1584 | 10.43% (9.72 - 11.15) | 22.98% (19.86 - 26.09) | 23.03% (19.65 - 26.4) | 19.25% (15.04 - 23.45) |
|  | Unemployed | 818 | 4.51% (4.1 - 4.91) | 17.53% (14.29 - 20.77) | 17.13% (13.63 - 20.64) | 11.89% (7.52 - 16.27) |
|  | Unpaid job | 8098 | 46.69% (45.6 - 47.78) | 22.68% (21.43 - 23.93) | 22.71% (21.35 - 24.06) | 18.84% (17.16 - 20.53) |
| Marital status |  |  |  |  |  |  |
|  | Single | 1312 | 8.45% (7.81 - 9.09) | 13.51% (11.14 - 15.88) | 12.78% (10.22 - 15.35) | 6.47% (3.27 - 9.67) |
|  | Married | 13825 | 82.46% (81.61 - 83.32) | 21.18% (20.23 - 22.13) | 21.08% (20.05 - 22.11) | 16.82% (15.54 - 18.1) |
|  | Divorced/separate from partner | 362 | 2.43% (2.06 - 2.79) | 16.68% (12.27 - 21.1) | 16.21% (11.44 - 21) | 10.75% (4.79 - 16.71) |
|  | Widow | 1111 | 6.66% (6.13 - 7.18) | 26.35% (22.8 - 29.9) | 26.68% (22.84 - 30.52) | 23.8% (19.01 - 28.59) |
| Quality of life |  |  |  |  |  |  |
|  | 1 | 3850 | 21.93% (21.06 - 22.8) | 22.55% (20.75 - 24.36) | 22.56% (20.62 - 24.52) | 18.67% (16.24 - 21.11) |
|  | 2 | 4031 | 24.36% (23.39 - 25.32) | 21.31% (19.53 - 23.1) | 21.22% (19.3 - 23.16) | 17% (14.59 - 19.41) |
|  | 3 | 3753 | 23.18% (22.26 - 24.1) | 20.97% (19.19 - 22.75) | 20.85% (18.93 - 22.78) | 16.54% (14.13 - 18.94) |
|  | 4 | 2543 | 17.1% (16.21 - 17.99) | 18.62% (16.58 - 20.66) | 18.31% (16.1 - 20.52) | 13.36% (10.61 - 16.12) |
|  | 5 | 2345 | 13.44% (12.74 - 14.13) | 18.67% (16.63 - 20.7) | 18.37% (16.16 - 20.56) | 13.43% (10.68 - 16.17) |
| Wealth index |  |  |  |  |  |  |
|  | Poorest | 3419 | 19.57% (18.7 - 20.44) | 18.62% (16.88 - 20.36) | 18.31% (16.43 - 20.19) | 13.36% (11.02 - 15.71) |
|  | Lower middle | 3049 | 20.06% (19.14 - 20.97) | 22.18% (20.18 - 24.18) | 22.16% (20 - 24.33) | 18.17% (15.47 - 20.87) |
|  | Middle | 3395 | 19.01% (18.22 - 19.79) | 21.97% (20.22 - 23.73) | 21.94% (20.04 - 23.84) | 17.89% (15.52 - 20.26) |
|  | Upper middle | 3167 | 20% (19.15 - 20.85) | 22.81% (20.81 - 24.8) | 22.85% (20.68 - 25) | 19.02% (16.32 - 21.71) |
|  | Richest | 2725 | 21.37% (20.33 - 22.41) | 18.56% (16.36 - 20.77) | 18.25% (15.87 - 20.64) | 13.28% (10.31 - 16.27) |
| Comorbidities |  |  |  |  |  |  |
| Diabetes Mellitus |  | 2251 | 14.22% (13.46 - 14.99) | 25.06% (22.67 - 27.44) | 25.28% (22.69 - 27.86) | 22.06% (18.83 - 25.27) |
| Hypertension |  | 6102 | 35.68% (34.62 - 36.73) | 24.67% (23.14 - 26.2) | 24.86% (23.2 - 26.52) | 21.53% (19.47 - 23.6) |
| Hypercholesterolemia |  | 5096 | 30.61% (29.59 - 31.62) | 24.47% (22.81 - 26.13) | 24.64% (22.85 - 26.44) | 21.26% (19.02 - 23.5) |
| MI (ever) |  | 1237 | 7.7% (7.11 - 8.28) | 25.11% (21.79 - 28.43) | 25.34% (21.74 - 28.93) | 22.12% (17.64 - 26.61) |
| MI (last year) |  | 325 | 2.16% (1.8 - 2.51) | 26.51% (19.56 - 33.46) | 26.85% (19.33 - 34.37) | 24.01% (14.63 - 33.4) |
| Lung disease (ever) |  | 836 | 4.87% (4.43 - 5.31) | 24.3% (20.6 - 28) | 24.46% (20.45 - 28.46) | 21.03% (16.04 - 26.03) |
| Lung disease (last year) |  | 473 | 2.84% (2.49 - 3.19) | 24.62% (19.61 - 29.64) | 24.81% (19.38 - 30.24) | 21.46% (14.7 - 28.24) |
| Stroke (ever) |  | 249 | 1.43% (1.19 - 1.67) | 27.6% (20.27 - 34.93) | 28.03% (20.1 - 35.96) | 25.49% (15.59 - 35.38) |
| Stroke (last year) |  | 43 | 0.22% (0.13 - 0.31) | 23.87% (9.59 - 38.16) | 23.99% (8.54 - 39.46) | 20.45% (1.17 - 39.74) |
| Cancer (last year) |  | 217 | 1.36% (1.12 - 1.6) | 17.26% (10.65 - 23.88) | 16.84% (9.69 - 24) | 11.53% (2.61 - 20.46) |
| Kidney disease |  | 2019 | 12.05% (11.31 - 12.79) | 25.85% (23.19 - 28.52) | 26.14% (23.26 - 29.03) | 23.12% (19.53 - 26.73) |
| Behavioral factors |  |  |  |  |  |  |
| Current cigarette smoker | Yes | 1492 | 9.51% (8.83 - 10.18) | 12.57% (10.06 - 15.08) | 11.76% (9.05 - 14.48) | 5.2% (1.81 - 8.59) |
| Second-hand smoker | Yes | 4639 | 30.6% (29.57 - 31.64) | 21.91% (20.24 - 23.58) | 21.87% (20.06 - 23.68) | 17.81% (15.55 - 20.06) |
| Physical activity (MET) | Yes | 7435 | 51.01% (49.85 - 52.17) | 21.09% (19.85 - 22.34) | 20.98% (19.64 - 22.34) | 16.7% (15.02 - 18.39) |
| Toothbrush | Yes | 13095 | 82.15% (81.39 - 82.91) | 20.25% (19.29 - 21.21) | 20.08% (19.04 - 21.11) | 15.56% (14.27 - 16.86) |
| Dental floss | Yes | 3107 | 21.87% (20.89 - 22.85) | 19.81% (17.74 - 21.88) | 19.6% (17.36 - 21.84) | 14.97% (12.18 - 17.76) |
| No oral hygiene | Yes | 2946 | 14.84% (14.13 - 15.54) | 23.08% (21.11 - 25.05) | 23.14% (21.01 - 25.27) | 19.38% (16.73 - 22.04) |
| Alcohol consumption frequency |  |  |  |  |  |  |
|  | Never | 15969 | 96.56% (96.11 - 97.01) | 20.88% (20.01 - 21.75) | 20.76% (19.82 - 21.7) | 16.41% (15.24 - 17.59) |
|  | Daily | 14 | 0.06% (0.02 - 0.1) | 5.37% (0- 15.95) | 3.97% (0 - 15.42) | -4.52% (-18.79 - 9.76) |
|  | Weekly | 75 | 0.51% (0.38 - 0.64) | 14.24% (6.13 - 22.35) | 13.57% (4.79 - 22.35) | 7.45% (0 - 18.4) |
|  | Monthly | 149 | 1.23% (0.92 - 1.55) | 17.48% (7.63 - 27.32) | 17.08% (6.42 - 27.73) | 11.83% (0 - 25.11) |
|  | Seasonal | 104 | 0.71% (0.47 - 0.95) | 12.92% (5.21 - 20.62) | 12.14% (3.8 - 20.48) | 5.67% (0 - 16.06) |
|  | 6 months or more | 153 | 0.93% (0.75 - 1.1) | 24.39% (16 - 32.78) | 24.56% (15.48 - 33.64) | 21.15% (9.83 - 32.48) |
| BMI |  |  |  |  |  |  |
|  | Underweight | 426 | 2.18% (1.92 - 2.44) | 9.15% (6.17 - 12.12) | 8.06% (4.84 - 11.28) | 0.58% (0 - 4.59) |
|  | Normal | 5169 | 30.91% (29.9 - 31.91) | 17.57% (16.13 - 19) | 17.18% (15.62 - 18.72) | 11.95% (10 - 13.88) |
|  | Overweight | 6447 | 39.73% (38.65 - 40.81) | 21.43% (20.01 - 22.85) | 21.35% (19.82 - 22.89) | 17.16% (15.24 - 19.07) |
|  | Obesity | 4495 | 27.18% (26.2 - 28.15) | 24.24% (22.55 - 25.93) | 24.39% (22.56 - 26.22) | 20.95% (18.67 - 23.23) |
| Covid-19 safety protocols |  |  |  |  |  |  |
| Mask |  | 15192 | 94.39% (93.95 - 94.84) | 20.73% (19.84 - 21.63) | 20.6% (19.63 - 21.57) | 16.21% (15.01 - 17.43) |
| Gloves |  | 5522 | 36.01% (34.92 - 37.1) | 21.12% (19.56 - 22.69) | 21.02% (19.33 - 22.72) | 16.74% (14.63 - 18.86) |
| Face shield |  | 621 | 5.77% (5.12 - 6.42) | 13.97% (10.68 - 17.25) | 13.28% (9.72 - 16.83) | 7.09% (2.65 - 11.51) |
| Washing hands |  | 14732 | 90.96% (90.32 - 91.61) | 20.81% (19.9 - 21.71) | 20.68% (19.7 - 21.66) | 16.32% (15.09 - 17.54) |
| Using public transport |  | 4212 | 25.15% (24.21 - 26.09) | 21.75% (20.11 - 23.4) | 21.7% (19.92 - 23.48) | 17.59% (15.38 - 19.82) |
| Having children attending school |  | 1925 | 9.43% (8.89 - 9.97) | 19.85% (17.55 - 22.15) | 19.64% (17.15 - 22.13) | 15.02% (11.92 - 18.13) |
| Number of daily contacts |  |  |  |  |  |  |
|  | under 10 | 11988 | 73.82% (72.84 - 74.8) | 21.02% (20 - 22.04) | 20.91% (19.81 - 22.01) | 16.6% (15.23 - 17.98) |
|  | 10 to 30 | 2884 | 17.9% (17.04 - 18.77) | 20.07% (18.11 - 22.03) | 19.88% (17.76 - 22) | 15.32% (12.68 - 17.97) |
|  | 30 to 50 | 691 | 4.27% (3.84 - 4.7) | 18.25% (14.63 - 21.87) | 17.91% (13.99 - 21.83) | 12.86% (7.98 - 17.75) |
|  | 50 and more | 648 | 4.01% (3.59 - 4.42) | 23.31% (18.99 - 27.63) | 23.39% (18.71 - 28.06) | 19.69% (13.86 - 25.53) |

**Table S3. The OR of the study variables and IgG seropositivity, crude and adjusted by age, sex, wealth index, and years of education**

| Variable | Status | Crude OR | Crude P value | Adjusted OR | Adjusted P value |
| --- | --- | --- | --- | --- | --- |
| Socio-demographic |  |  |  |  |  |
| Age (reference: 25-29) | 30-39 | 1.096(0.883,1.361) | 0.404 | 1.012(0.811,1.263) | 0.917 |
|  | 40-49 | 1.414(1.142,1.75) | 0.002 | 1.261(1.006,1.58) | 0.044 |
|  | 50-59 | 1.747(1.412,2.162) | <0.001 | 1.5(1.192,1.887) | 0.001 |
|  | 60 and above | 1.951(1.58,2.408) | <0.001 | 1.599(1.251,2.045) | <0.001 |
| Sex (reference: female) | Male | 0.851(0.766,0.945) | 0.003 | 0.867(0.775,0.969) | 0.012 |
| Education (reference: 0 years) | 1 to 7 y | 0.766(0.666,0.881) | <0.001 | 0.772(0.659,0.904) | 0.001 |
|  | 7 to 12 y | 0.638(0.544,0.748) | <0.001 | 0.715(0.586,0.874) | 0.001 |
|  | 12+y | 0.552(0.477,0.639) | <0.001 | 0.651(0.533,0.793) | <0.001 |
| Occupation (reference: unemployed) | Freelancer | 1.091(0.851,1.398) | 0.491 | 1.151(0.893,1.484) | 0.279 |
|  | Private sector worker | 0.884(0.637,1.228) | 0.463 | 1.038(0.744,1.449) | 0.825 |
|  | Public sector worker | 1.015(0.748,1.377) | 0.923 | 1.151(0.835,1.588) | 0.391 |
|  | Retired | 1.404(1.055,1.866) | 0.02 | 1.182(0.872,1.604) | 0.281 |
|  | Unpaid job | 1.38(1.091,1.746) | 0.007 | 1.279(0.955,1.714) | 0.099 |
| Marital status (reference: married) | Single | 0.581(0.471,0.717) | <0.001 | 0.747(0.593,0.942) | 0.014 |
|  | Divorced/separated with partner | 0.745(0.54,1.029) | 0.074 | 0.837(0.602,1.165) | 0.292 |
|  | Widow | 1.331(1.099,1.613) | 0.003 | 1.005(0.806,1.252) | 0.967 |
| Quality of life (reference: level 1) | 2 | 0.93(0.802,1.079) | 0.339 | 0.999(0.857,1.166) | 0.992 |
|  | 3 | 0.911(0.785,1.058) | 0.222 | 1.018(0.872,1.188) | 0.824 |
|  | 4 | 0.786(0.663,0.931) | 0.005 | 0.937(0.785,1.12) | 0.477 |
|  | 5 | 0.788(0.665,0.934) | 0.006 | 0.928(0.777,1.108) | 0.408 |
| Wealth index (reference: low) | Low-middle | 1.246(1.058,1.466) | 0.008 | 1.348(1.144,1.588) | <0.001 |
|  | Middle | 1.231(1.055,1.436) | 0.008 | 1.372(1.172,1.607) | <0.001 |
|  | Upper-middle | 1.292(1.099,1.518) | 0.002 | 1.534(1.299,1.812) | <0.001 |
|  | Richest | 0.996(0.828,1.199) | 0.97 | 1.228(1.01,1.493) | 0.039 |
| Comorbidities |  |  |  |  |  |
| Diabetes Mellitus |  | 1.334(1.161,1.534) | <0.001 | 1.104(0.949,1.283) | 0.199 |
| Hypertension |  | 1.437(1.292,1.598) | <0.001 | 1.143(1.004,1.3) | 0.043 |
| Hypercholesterolemia |  | 1.372(1.229,1.532) | <0.001 | 1.179(1.042,1.334) | 0.009 |
| MI (ever) |  | 1.31(1.089,1.576) | 0.004 | 1.047(0.856,1.281) | 0.653 |
| MI (last year) |  | 1.389(0.969,1.993) | 0.074 | 1.12(0.765,1.64) | 0.559 |
| Lung disease (ever) |  | 1.239(1.006,1.526) | 0.044 | 1.083(0.876,1.34) | 0.461 |
| Lung disease (last year) |  | 1.256(0.953,1.653) | 0.105 | 1.08(0.82,1.423) | 0.584 |
| Stroke (ever) |  | 1.466(1.012,2.123) | 0.043 | 1.231(0.832,1.821) | 0.298 |
| Stroke (last year) |  | 1.199(0.546,2.634) | 0.652 | 1.063(0.507,2.231) | 0.872 |
| Cancer (last year) |  | 0.794(0.498,1.265) | 0.332 | 0.74(0.453,1.208) | 0.228 |
| Kidney disease |  | 1.388(1.195,1.612) | <0.001 | 1.14(0.966,1.346) | 0.12 |
| Behavioral factors |  |  |  |  |  |
| Current cigarette smoker |  | 0.521(0.412,0.659) | <0.001 | 0.523(0.407,0.672) | <0.001 |
| Second-hand smoker |  | 1.017(0.904,1.144) | 0.775 | 1.045(0.92,1.187) | 0.496 |
| Physical activity (MET) |  | 1.011(0.907,1.127) | 0.845 | 0.944(0.841,1.059) | 0.325 |
| Toothbrush |  | 0.843(0.749,0.95) | 0.005 | 0.977(0.853,1.12) | 0.739 |
| Dental floss |  | 0.927(0.805,1.068) | 0.296 | 1.066(0.912,1.246) | 0.42 |
| No oral hygiene |  | 1.174(1.035,1.33) | 0.012 | 1.012(0.88,1.164) | 0.865 |
| Alcohol consumption frequency (reference: never) | Daily | 0.215(0.027,1.721) | 0.148 | 0.237(0.028,2.035) | 0.19 |
|  | Weekly | 0.629(0.323,1.224) | 0.173 | 0.695(0.346,1.396) | 0.306 |
|  | Monthly | 0.802(0.405,1.59) | 0.529 | 1.031(0.498,2.138) | 0.934 |
|  | Seasonal | 0.562(0.283,1.117) | 0.1 | 0.654(0.321,1.331) | 0.241 |
|  | 6 months or more | 1.222(0.773,1.932) | 0.39 | 1.53(0.957,2.447) | 0.076 |
| BMI (reference: normal) | Underweight | 0.472(0.326,0.685) | <0.001 | 0.511(0.35,0.746) | 0.001 |
| BMI | Overweight | 1.28(1.124,1.458) | <0.001 | 1.25(1.09,1.434) | 0.001 |
| BMI | Obesity | 1.502(1.311,1.719) | <0.001 | 1.346(1.166,1.555) | <0.001 |
| COVID-19 safety protocols |  |  |  |  |  |
| Mask |  | 0.908(0.738,1.117) | 0.362 | 0.89(0.718,1.103) | 0.288 |
| Gloves |  | 1.028(0.919,1.151) | 0.626 | 1.028(0.916,1.154) | 0.638 |
| Face shield |  | 0.602(0.456,0.795) | <0.001 | 0.687(0.514,0.917) | 0.011 |
| Washing hands |  | 0.987(0.818,1.192) | 0.892 | 0.962(0.794,1.164) | 0.689 |
| Public transport |  | 1.077(0.96,1.208) | 0.206 | 1.055(0.932,1.195) | 0.394 |
| Children attending school |  | 0.936(0.801,1.092) | 0.4 | 0.984(0.83,1.165) | 0.849 |
| Number of daily contacts (reference: under 10) | 10 to 30 | 0.943(0.823,1.082) | 0.404 | 1.067(0.921,1.236) | 0.388 |
|  | 30 to 50 | 0.839(0.653,1.077) | 0.168 | 1.031(0.791,1.345) | 0.819 |
|  | 50 and more | 1.142(0.89,1.465) | 0.297 | 1.447(1.11,1.887) | 0.006 |

**Table S4. The OR of the study variables and history of COVID-19 infection based on claims, crude and adjusted by age, sex, wealth index, and years of education**

| Variable | Status | Crude OR | Crude P value | Adjusted OR | Adjusted P value |
| --- | --- | --- | --- | --- | --- |
| Socio-demographic |  |  |  |  |  |
|  |  |  |  |  |  |
| Age (reference: 25-29) | 30-39 | 1.306(0.974,1.751) | 0.075 | 1.274(0.942,1.723) | 0.117 |
|  | 40-49 | 1.26(0.944,1.683) | 0.117 | 1.35(0.997,1.828) | 0.052 |
|  | 50-59 | 1.338(0.999,1.792) | 0.051 | 1.457(1.067,1.99) | 0.018 |
|  | 60 and above | 1.258(0.94,1.684) | 0.122 | 1.564(1.134,2.157) | 0.006 |
| Sex (reference: female) | Male | 0.852(0.733,0.989) | 0.036 | 0.809(0.689,0.949) | 0.009 |
| Education (reference: 0 years) | 1 to 7 y | 1.135(0.903,1.427) | 0.278 | 1.082(0.846,1.385) | 0.53 |
|  | 7 to 12 y | 1.075(0.825,1.4) | 0.593 | 1.112(0.833,1.486) | 0.472 |
|  | 12+y | 1.626(1.321,2.002) | <0.001 | 1.586(1.237,2.034) | <0.001 |
| Occupation (reference: unemployed) | Freelancer | 1.351(0.923,1.976) | 0.122 | 1.278(0.859,1.902) | 0.227 |
|  | Private sector worker | 1.619(1.016,2.579) | 0.043 | 1.442(0.893,2.327) | 0.134 |
|  | Public sector worker | 3(1.984,4.538) | <0.001 | 2.147(1.371,3.361) | 0.001 |
|  | Retired | 1.562(1.045,2.336) | 0.03 | 1.066(0.685,1.658) | 0.777 |
|  | Unpaid job | 1.539(1.081,2.192) | 0.017 | 1.078(0.713,1.632) | 0.721 |
| Marital status (reference: married) | Single | 0.78(0.601,1.013) | 0.062 | 0.789(0.596,1.045) | 0.099 |
|  | Divorced/separate with partner | 0.686(0.438,1.077) | 0.102 | 0.752(0.471,1.202) | 0.234 |
|  | Widow | 0.856(0.638,1.149) | 0.301 | 0.979(0.707,1.355) | 0.899 |
| Quality of life (reference: level 1) | 2 | 0.917(0.745,1.13) | 0.417 | 0.83(0.67,1.029) | 0.089 |
|  | 3 | 0.848(0.695,1.034) | 0.103 | 0.792(0.642,0.976) | 0.029 |
|  | 4 | 0.941(0.736,1.204) | 0.629 | 0.829(0.638,1.076) | 0.158 |
|  | 5 | 0.708(0.558,0.899) | 0.005 | 0.67(0.519,0.864) | 0.002 |
| Wealth index (reference: low) | Low-middle | 1.682(1.299,2.176) | <0.001 | 1.556(1.201,2.017) | 0.001 |
|  | Middle | 1.772(1.41,2.226) | <0.001 | 1.699(1.345,2.145) | <0.001 |
|  | Upper-middle | 2.257(1.803,2.826) | <0.001 | 2.058(1.637,2.587) | <0.001 |
|  | Richest | 2.408(1.897,3.057) | <0.001 | 1.981(1.53,2.565) | <0.001 |
| Comorbidities |  |  |  |  |  |
| Diabetes Mellitus | Yes | 1.327(1.093,1.612) | 0.004 | 1.311(1.062,1.62) | 0.012 |
| Hypertension | Yes | 1.125(0.967,1.309) | 0.128 | 1.139(0.931,1.393) | 0.208 |
| Hypercholesterolemia | Yes | 1.2(1.028,1.401) | 0.021 | 1.184(0.993,1.411) | 0.06 |
| MI (ever) | Yes | 1.476(1.158,1.882) | 0.002 | 1.536(1.182,1.996) | 0.001 |
| MI (last year) | Yes | 1.308(0.833,2.054) | 0.243 | 1.418(0.878,2.29) | 0.153 |
| Lung disease (ever) | Yes | 1.915(1.479,2.48) | <0.001 | 1.935(1.484,2.523) | <0.001 |
| Lung disease (last year) | Yes | 1.515(1.078,2.13) | 0.017 | 1.442(1.029,2.02) | 0.034 |
| Stroke (ever) | Yes | 1.198(0.677,2.12) | 0.535 | 1.312(0.734,2.344) | 0.36 |
| Stroke (last year) | Yes | 2.444(0.56,10.663) | 0.235 | 2.473(0.603,10.144) | 0.209 |
| Cancer (last year) | Yes | 1.199(0.611,2.352) | 0.599 | 1.239(0.628,2.443) | 0.537 |
| Kidney disease | Yes | 0.944(0.767,1.161) | 0.583 | 1.01(0.805,1.266) | 0.934 |
| Behavioral factors |  |  |  |  |  |
| Current cigarette smoker | Yes | 0.537(0.375,0.77) | 0.001 | 0.592(0.407,0.86) | 0.006 |
| Second-hand smoker | Yes | 0.918(0.778,1.083) | 0.31 | 1(0.84,1.19) | 0.997 |
| Physical activity (MET) | Yes | 1.046(0.899,1.217) | 0.56 | 0.999(0.852,1.171) | 0.987 |
| Toothbrush | Yes | 1.417(1.168,1.719) | <0.001 | 1.28(1.033,1.586) | 0.024 |
| Dental floss | Yes | 1.625(1.367,1.931) | <0.001 | 1.404(1.154,1.708) | 0.001 |
| No oral hygiene | Yes | 0.609(0.503,0.739) | <0.001 | 0.748(0.61,0.917) | 0.005 |
| Alcohol consumption frequency (reference: never) | Daily | 0.273(0.034,2.176) | 0.22 | 0.372(0.047,2.958) | 0.35 |
|  | Weekly | 0.452(0.124,1.649) | 0.229 | 0.451(0.124,1.635) | 0.226 |
|  | Monthly | 1.121(0.44,2.854) | 0.811 | 1.199(0.471,3.047) | 0.704 |
|  | Seasonal | 1.011(0.464,2.206) | 0.978 | 1.125(0.51,2.482) | 0.77 |
|  | 6 months or more | 0.717(0.347,1.481) | 0.369 | 0.819(0.396,1.697) | 0.592 |
| BMI (reference: normal) | Underweight | 0.764(0.362,1.613) | 0.481 | 1.055(0.502,2.218) | 0.887 |
|  | Overweight | 1.27(1.056,1.527) | 0.011 | 1.193(0.987,1.442) | 0.069 |
|  | Obesity | 1.533(1.268,1.853) | <0.001 | 1.439(1.177,1.758) | <0.001 |
| COVID-19 safety protocols |  |  |  |  |  |
| Mask | Yes | 2.378(1.55,3.649) | <0.001 | 2.197(1.466,3.292) | <0.001 |
| Gloves | Yes | 1.012(0.868,1.179) | 0.881 | 0.962(0.823,1.124) | 0.624 |
| Shield | Yes | 1.089(0.76,1.561) | 0.642 | 1.145(0.788,1.663) | 0.477 |
| Washing hands | Yes | 1.497(1.1,2.036) | 0.01 | 1.384(0.997,1.921) | 0.052 |
| transport | Yes | 0.977(0.818,1.167) | 0.8 | 1.037(0.852,1.262) | 0.717 |
| Children attending school | Yes | 0.663(0.514,0.855) | 0.002 | 0.63(0.475,0.837) | 0.001 |
| Number of daily contacts (reference: under 10) | 10 to 30 | 1.329(1.093,1.615) | 0.004 | 1.454(1.157,1.826) | 0.001 |
|  | 30 to 50 | 1.251(0.872,1.796) | 0.225 | 1.378(0.937,2.028) | 0.104 |
|  | 50 and more | 1.399(1.019,1.921) | 0.038 | 1.582(1.123,2.227) | 0.009 |

**References**

1. Poustchi H, Darvishian M, Mohammadi Z, Shayanrad A, Delavari A, Bahadorimonfared A, et al. SARS-CoV-2 antibody seroprevalence in the general population and high-risk occupational groups across 18 cities in Iran: a population-based cross-sectional study. Lancet Infect Dis [Internet]. 2021 Apr 1 [cited 2022 Jan 7];21(4):473–81. Available from: https://pubmed.ncbi.nlm.nih.gov/33338441/

2. Zare F, Ameri H, Madadizadeh F, Aghaei MR. Validity and reliability of the EQ-5D-3L (a generic preference-based instrument used for calculating quality-adjusted life -years) for patients with type 2 diabetes in Iran. Diabetes Metab Syndr Clin Res Rev. 2021 Jan 1;15(1):319–24.

3. Mohebi F, Mohajer B, Yoosefi M, Sheidaei A, Zokaei H, Damerchilu B, et al. Physical activity profile of the Iranian population: STEPS survey, 2016. BMC Public Health [Internet]. 2019 [cited 2022 Apr 20];19(1). Available from: https://pubmed.ncbi.nlm.nih.gov/31519165/
